# Gender and Alcoholic Subtypes

**Published:** 1996

**Authors:** Frances K. Del Boca, Michie N. Hesselbrock

**Affiliations:** Frances K. Del Boca, Ph.D., formerly an assistant professor in the Department of Psychiatry, University of Connecticut Medical School, Farmington, Connecticut, is now at the Psychology Department, University of South Florida, Tampa, Florida. Michie N. Hesselbrock, Ph.D., is a professor at the University of Connecticut School of Social Work, West Hartford, Connecticut

**Keywords:** AOD dependence, disorder classification, gender differences, disease severity, etiology, comorbidity, emotional and psychiatric depression, antisocial personality disorder, anxiety state, intervention, patient-treatment matching, research

## Abstract

Although women generally have been subjects of alcohol research less often than men, gender can be used as a defining characteristic in subtyping schemes. Whether the sexes actually differ in aspects of their alcoholism, such as in etiology and degree of severity, however, is not known. Analyzing a sample of male and female alcoholics using several different statistical methods, the researchers found that men and women with either a severe or mild form of alcoholism differed little in their character profiles and etiologies. Women and men with moderately severe alcoholism, however, tended to differ with respect to co-occurring psychopathologies (e.g., depression or antisocial personality) and the degree to which they drank to relieve other conditions (e.g., boredom). These findings suggest that different forms of alcoholism treatment may be most effective for men and women with moderately severe alcoholism. However, as is the case with any subtyping scheme, this conclusion cannot be applied to the general population without further research.

Researchers and practitioners have long recognized that alcoholics^1^ are not all alike; rather, they vary along numerous dimensions, including their family histories, their stated reasons for drinking, and their personality characteristics. These differences have important implications for understanding the etiology (i.e., development) of alcoholism as well as for prevention, intervention, and treatment efforts. Subtyping—that is, dividing a large group of alcoholics into smaller groups of alcoholics with similar characteristics—is one method of bringing order to the variability within this population.

In general, women have been studied less often than men in all areas of alcohol research. Nevertheless, scientists have found that differences exist between male and female alcoholics, and gender has surfaced as a defining characteristic in many major subtyping schemes. By studying gender differences among alcoholics, researchers have compiled traits and circumstances that tend to be more common for men or for women (see Lex 1990). Subtyping formulations potentially can provide a simple way to summarize gender-related similarities and differences among alcoholics.

This article presents an overview of research and theory on gender in relation to alcoholism subtyping. Five major questions are addressed:

- How have researchers approached the study of gender in relation to alcoholic subtypes?
- In terms of subtypes, what are the important similarities and differences between the sexes?
- How can subtyping research add to the understanding of how men and women differ in their development of alcohol problems?
- How might findings from subtyping research affect intervention and treatment for men and women?
- What are the limitations of existing subtyping research?

## Gender in Subtype Formulations

The content of subtype profiles often depends on the range of attributes that researchers decide to consider when making distinctions among alcoholics. Two prominent subtyping studies (Schuckit et al. 1969; Cloninger 1987) brought gender differences into consideration and expanded on earlier typological approaches that had tended to emphasize differences in drinking patterns.

### Early Subtypes

In one of the earliest studies of gender and alcoholism subtypes, Schuckit and his colleagues (1969) described two major variations in female alcoholism, *primary* and *secondary.* Primary alcoholics were women whose damaging drinking patterns developed apart from any other psychological disorders, and secondary alcoholics were women for whom alcoholism developed after they experienced either depression or anxiety (i.e., affective disorders). This distinction, which has become somewhat controversial, is consistent with numerous studies that have found depression and anxiety to be more prevalent in female than in male alcoholics (for review, see Hesselbrock 1991). Schuckit’s scheme was the first to focus attention on women and concomitant mood disorder (e.g., affective disorder).

In what was to become one of the most influential approaches to subtyping alcoholics, Cloninger (1987) proposed a subclassification of two types: type II (“male-limited”) alcoholics, who experienced a more severe form of alcohol abuse and had fathers who exhibited both criminal behavior and severe alcohol abuse, and type I (“milieu-limited”) alcoholics, whose less severe alcoholism was less often associated with family history. Bohman and colleagues (1981) analyzed women from the Cloninger study and placed women in a single category, type I. This partitioning was based on differences in the alcoholics’ temperaments (e.g., whether their actions were driven by their desire for reward or avoidance of harm) rather than their psychopathology. In addition to drawing attention to alcoholic men, Cloninger’s work was among the first to consider the genetic contribution in alcoholism subtyping. Type II was characterized by genetic factors independent of environmental influences, whereas type I was influenced by both genetic and social/situational factors (e.g., social norms regarding drinking). Historically, differing social prescriptions for drinking have existed for each gender, wherein more sanctions have been applied to women than to men. Thus, Cloninger’s recognition of the potential importance of nongenetic factors in the etiology of alcoholic subtypes is likely to have implications for understanding the differential development of alcohol problems in both women and men.

### The Type A-Type B Subtype

Researchers have arrived at another classification—the type A-type B subtype—that addresses alcoholism in both men and women. Type A and type B alcoholics are defined in terms of a broad range of factors, including psychopathology and family history of alcoholism, and are grouped according to the severity of their disorder. These two subtypes were derived from studies using a statistical method (i.e., cluster analysis) that classifies individuals into groups (i.e., clusters) based on their similarities with regard to the attributes selected for analysis (Babor et al. 1992). In the original typology study, 17 different characteristics, designed to reflect four broad domains (i.e., premorbid risk factors, alcohol and other drug [AOD] use, chronic nature and consequences of alcohol use, and psychiatric symptoms), were used to classify a large and diverse group of inpatients (228 men and 85 women) who met the American Psychiatric Association’s *Diagnostic and Statistical Manual, Third Edition* (DSM–III) criteria for alcohol dependence (see table 1). Study participants were recruited from three treatment facilities more than a decade ago, and intensive followup evaluations were performed both 1 year and 3 years after the initial treatment.

**Figure:**
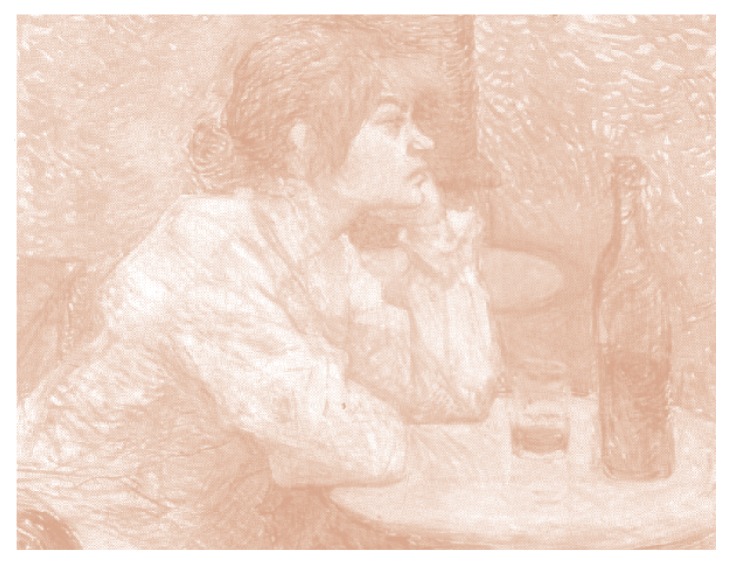
One variety of female alcoholism illustrated in “The Hangover,” 1887–1889, by Henri de Toulouse-Lautrec. Reproduced with permisson from the Fogg Art Museum, Harvard University Art Museums, © President and Fellows, Harvard College, Harvard University Art Museums.

#### The Risk-Severity Distinction

Analyses produced a useful set of two subtypes (type A and type B) that differed significantly in terms of 16 of the 17 attributes. Type A alcoholics were characterized as having a low risk for developing alcoholism; those who did develop the disease did so at a low level of severity. In contrast, type B alcoholics had more risk factors, such as a family history of alcoholism, a younger age of onset, and early conduct problems (e.g., getting into fights or stealing). Type B alcoholics also exhibited more severe dependence symptoms, such as tolerance and withdrawal; alcohol-related consequences, including liver disease and job loss; and psychopathologies, such as depression and anxiety. Separate analyses for each sex produced the basic type A-type B distinction (see Babor et al. 1992). Alcoholic men were disproportionately categorized, however, as type B (53 percent of the men compared with 38 percent of the women), whereas women were more likely to be classified as type A (62 percent of the females versus 47 percent of the males).

The type A-type B typology has been replicated in several studies using different samples and somewhat different measures (Litt et al. 1992; Brown et al. 1994; Schuckit et al. 1995). In addition, researchers consistently have found that males and females differ in their distribution among the two subtypes: Males are more prominent within the type B category (e.g., Brown et al. 1994; Schuckit et al. 1995).

The two subtypes also have proven useful for predicting which group has the best chance of recovery from alcoholism. Type A alcoholics generally exhibit better outcomes (Babor et al. 1992). Finally, and perhaps most important from a practical perspective, this typology may help clinicians match patients to specific treatments that will be the most effective for their type of alcoholism (Litt et al. 1992) (for an update of findings regarding the type A-type B distinction, see the article by Allen, pp. 24–29). The type A-type B distinction implies quantitative, as well as qualitative, differences among alcoholics. That is, in addition to indicating that two categories of alcoholics exist with characteristic profiles, this formulation suggests that alcoholics differ along a general dimension or continuum characterized as low risk-low severity at one end and high risk-high severity at the other. Several other investigations have confirmed this finding (e.g., Goodwin et al. 1994). Women and men may simply fall at different extremes along this dimension, or important differences may exist between the genders in terms of subtype profiles.

As demonstrated by the type A-type B typology, when researchers consider a broad range of attributes while subtyping alcoholics, they obtain categories that apply to both genders. These subtypes are primarily distinguished in terms of risk for and severity of alcoholism. At the same time, however, the number of women or men within each subtype, together with the results of research comparing alcoholic men and women, suggests that important gender differences may exist within each subtype. These differences may be specific characteristics that have etiologic and treatment significance for both men and women.

### Gender Similarities and Differences Within Each Subtype

When a study considers gender, a different subtyping solution may be reached. To examine more fully the interaction between gender and the type A-type B distinction, Del Boca (1994) conducted several secondary analyses using the data set from the typology study reviewed earlier (i.e., Babor et al. 1992). Men and women within each subtype were first compared using the subtype classifications produced in the original study by Babor (Del Boca 1994). The results of these comparisons, summarized in table 1, show that the profiles of type B males and females are relatively similar, whereas women and men categorized as type A differ.

As indicated in table 1, the type B men and the type B women differed significantly on only 4 of the 17 attributes used to define the typology. The men reported longer drinking histories, more severe lifetime consequences, and more symptoms of antisocial personality disorder^2^ than did the type B women; in contrast, women in this category showed significantly higher levels of a family history of alcoholism. Despite expected differences in body size and metabolism, the sexes did not differ in terms of the quantity of alcohol consumed or dependence severity, nor were there differences in symptoms of affective disorders.

In contrast, substantial gender differences were evident within the type A group for 13 of the 17 comparisons. On average, the type A male alcoholics reported that they began drinking at an earlier age and were found to have higher scores on a measure of personality that tended to distinguish alcoholics from nonalcoholics; however, as in the type B category, type A female alcoholics exhibited higher levels of family history of alcoholism. Men in this subtype reported drinking greater quantities of alcohol than their female counterparts but reported less drinking in response to stress and other negative states (e.g., feeling sad) and less use of tranquilizing drugs. Although the men had longer drinking histories, they appeared to suffer fewer physical and social consequences and to have fewer medical complaints than did the women. Perhaps most striking were the differences in psychopathology. As in the type B category, type A men exhibited more symptoms of antisocial personality; at the same time, however, the women showed significantly more depression and anxiety.

This comparison suggests two tentative conclusions regarding gender and alcoholic subtypes. First, it appears that the more severe form of alcoholism, type B, is not male limited, in contrast to Cloninger’s type II alcoholism. Although a smaller proportion of the women fell into this category, those who did may be comparable to the men in terms of a variety of risk and severity factors. Second, those men and women categorized as type A alcoholics appeared to differ in important ways, as previously described. Most notably, women in this group exhibited higher levels of affective disturbance (e.g., depression and anxiety); reported more severe medical and social consequences of their alcohol use; and appeared to self-medicate, using tranquilizers as well as alcohol (table 1).

### A Second Look at Gender and Subtypes

To further explore gender in relation to subtypes, additional analyses were performed on the Babor study data using the statistical techniques mentioned earlier (Del Boca and Hesselbrock 1995). Although the two-cluster solution subtypes (i.e., type A-type B) effectively represented the study sample in terms of risk and severity, new groups were derived to explore whether meaningful, gender-related subtypes would emerge. Using the new analysis, researchers discovered that dividing the sample into four clusters also produced a functional solution. Differences between the men and women in the new typology were most evident in two of the groups. Table 2 presents profiles of the four new subtypes suggested by this analysis.

The largest subtype, containing approximately one-third of the cases (28 percent of the men in the study population and 39 percent of the women), was characterized by relatively low risk and severity and was labeled as such. A second group contained equal proportions of men and women (22 percent from each group) and included those cases with the most pervasive family histories of alcoholism and the lowest age for first alcohol use. This subtype, labeled “high risk-high severity,” was characterized by conduct problems, illicit drug use, and antisocial personality.

The two intermediate subgroups were more gender specific. The “internalizing” subtype, which was labeled as such because of the ways in which its members expressed feelings and responded to their environments, included 32 percent of the women in the study population and only 11 percent of the men. This group comprised depressed and anxious alcoholics who reported often using alcohol to relieve anxiety or boredom (i.e., relief drinking). Members of this subtype were severely alcohol dependent and had medical or physical problems resulting from alcohol use. In addition, they showed only a moderate family history risk. The other group, the “externalizing” subtype, was predominantly male (containing 38 percent of the men versus 7 percent of the women), thereby indicating a gender bias. Members of this group also reported only a moderate family history of alcoholism. They did, however, report high levels of alcohol use, social consequences, and antisocial personality.

Members of the four groups also were compared in terms of age, and significant differences were found. Those in the high risk-high severity subtype were younger than those in the other three groups (mean age = 27 versus 42 years). Thus, the division of patients into the first group appears in part to have been related to their age and their drug-use practices (discussed below).

Comparisons among the four groups both 1 year and 3 years later revealed significant differences. For example, both the high risk-high severity group and the externalizing group tended to show poor outcomes relative to the other two subtypes. The two groups also were characterized by symptoms of antisocial personality disorder, which, not surprisingly, are associated with poor prognosis.

## Implications for Etiology

The four-group typology described above suggests that several pathways may lead to the development of alcohol problems in both women and men. These subtypes do not directly address the issue of etiology; nevertheless, with other research findings, they provide a basis for speculation about the course of alcoholism for different groups of women and men.

Consistent with recent research, the typology findings suggest that a family history of alcoholism predisposes women, as well as men, to a wide range of problems, one of which is alcoholism. The high risk-high severity group is characterized by high levels of risk factors, including a strong family history of alcoholism, that are evident at a relatively young age in both genders and result in similar characteristics for both men and women. As Hill and colleagues (1994) suggest, however, the progression toward alcohol problems may change, perhaps as a result of environmental factors (e.g., childhood upbringing). Such factors appear to dilute the effect of a family history of alcoholism on the later development of alcoholism. Thus, it is possible that differing childhood environments for males and females at risk for developing alcohol problems can alter whether and how they develop these problems later in life.

The two subtypes that appear to be gender related (i.e., internalizing and externalizing) share some characteristics; these attributes suggest that the etiologies among these groups may depend less on inherited characteristics than do the disease origins of the high risk-high severity subtype. The internalizing and externalizing groups are equivalent in terms of family history (i.e., both have moderate levels), degree of alcohol dependence, and alcohol-related consequences. The predominantly male, externalizing subtype, however, shows more early signs of problem behavior, including conduct problems, and earlier age of onset for problem drinking. In contrast, the internalizing subtype exhibits higher levels of depression, anxiety, and relief drinking. The characteristics are not entirely gender specific. They do, however, mirror gender differences—such as a higher incidence of depression and anxiety among women—commonly reported for nonalcoholic populations. The subtype differences also bear a clear correspondence to traditional gender roles (see Del Boca 1994), which reflect social norms about what is thought to be the “appropriate” behavior of men and women. Traditionally, the social “shoulds” for men within our culture emphasize assertive, task-oriented behavior, whereas those for women prescribe emotional expressiveness and deference. The extremes of these behaviors can result in the types of mood disorders characteristic of these two alcoholic subtypes.

Evidence from studies of adolescents supports differing etiologies for males and females, suggesting that adolescent girls who are more involved in AOD use than their peers have characteristics reminiscent of the internalizing subtype. Likewise, adolescent boys who use AOD’s have profiles similar to the externalizing subtype (Del Boca et al. 1995). Thus, young people’s responses to their environment, which appear to be connected to their gender, may predict their development of alcohol problems.

The moderate levels of early risk factors found in the two gender-related subtypes, together with the parallels to conventional gender roles, suggest that the development and expression of alcohol problems in the two groups depend on sociocultural factors (e.g., differing social expectations for women and men or differential approval of drinking by men versus women) rather than an inherited predisposition. This observation suggests an etiology for women in the internalizing group. Despite the current acceptability of a wider range of roles for women in American society, assertiveness among women is still discouraged. Thus, females might be expected to internalize problems and to self-medicate with alcohol to a greater degree than males; higher levels of negative affect and guilt as a result of drinking may set the stage for an alcohol consumption pattern that could result in alcoholism for the internalizing subtype. Similarly, the greater acceptability of aggressiveness and alcohol use among males may contribute to the pattern observed in the externalizing subtype.

## Considerations for Intervention and Treatment

Numerous articles have been published summarizing how women and men seek treatment for alcoholism and examining gender differences in treatment outcome (e.g., Stobar and Annis 1996; Jarvis 1992). In addition to these studies, subtyping research may be especially useful for designing intervention and treatment programs.

Some suggestions for alcoholism treatment that may be gleaned from prevention, treatment, and typology research are as follows:

- Both gender and the type A-type B distinction currently are under investigation as the potential bases for patient-treatment matching in Project MATCH, a large, multisite, clinical trial examining whether matching clients with certain attributes to particular treatment modalities improves outcome (see Project MATCH Research Group 1993). The results of this study will provide empirical evidence regarding the usefulness of treatment matching based on gender or on subtypes that distinguish people specifically on the basis of risk and severity. Although this straightforward approach may prove promising, the four-subtype formulation presented earlier suggests that matching may require a more complex strategy that considers gender, in combination with risk and severity, for effectively treating the whole range of the alcoholic population.
- The profiles of the most and least severe subtypes in the four-group scheme indicate more similarities than differences between the sexes in terms of treatment needs. For example, choice of a particular intervention or treatment could be based on the severity of alcohol problems evidenced by the individual. Low-intensity (e.g., outpatient) treatment might be used for those in the low risk-low severity group, whereas more intensive (e.g., day hospital or inpatient) treatment may be appropriate for the high risk-high severity group. Well-designed treatment-outcome research involving both female and male alcoholics is needed to empirically validate the use of specific treatment approaches for these two groups. Members of the internalizing and externalizing subtypes, however, differ in terms of their concomitant psychopathology and may differentially benefit from programs designed to address their range of symptoms. In these two groups, affective disorders should be addressed as comorbid diagnoses. For example, coping-skills training could be used to address depression and anxiety. As suggested by prior matching research, those in the externalizing group also may benefit more from coping-skills therapy than from less structured treatments, such as insight-oriented group therapy (Litt et al. 1992).^3^
- Other research proposes additional considerations in terms of addressing the different needs of women and men. Women may require more ancillary services (e.g., child care) and may prefer same-sex therapists, although the empirical evidence in support of this preference is mixed. Finally, other experiences and problems that may require special services are linked to gender but not addressed in the subtype literature. Alcoholic women, for example, are more likely than alcoholic men to report prior histories of physical and sexual abuse that could affect their success in treatment (e.g., Windle 1995).

### Prevention Considerations

In terms of prevention and early intervention, research suggests that female adolescents with AOD-related problems often are referred to treatment later than their male counterparts and at a time when their problems are more severe (Del Boca et al. 1995). In part, this delay may result from the tendency of people to presume that girls are less likely to become involved in AOD use. It also may stem, however, from the tendency of young women to internalize their problems, making their alcohol abuse much less visible than that of their male counterparts. Parents, teachers, and others who interact with adolescents should be trained to identify “internalizers” who are predisposed to alcohol-related problems (e.g., these adolescents often are withdrawn or moody) as well as those who have more obvious behavioral problems.

## Limitations of Existing Research

As evidenced by this issue of *Alcohol Health & Research World*, considerable ongoing research on alcoholic subtypes exists that has important practical as well as theoretical implications. Although research on alcoholic women has increased in recent years, the role of gender in relation to alcoholic subtyping is not often studied. Consequently, the implications of the analyses reported here are somewhat speculative. More research is needed to understand the manner in which genetic and sociocultural factors influence drinking patterns and the development of drinking problems in men and women.

In addition to the limitations of direct empirical evidence, certain caveats regarding subtyping research in general should be noted. The analytical methods used to identify subtypes (e.g., cluster analysis) are primarily descriptive in nature and intended to uncover typological structure in empirical data. Although the solutions that these methods produce can aid in the development of subtyping theory and inform the design of prevention and treatment programs, the results are primarily of heuristic (i.e., educational) value. The subtypes suggested by such methods reflect only the characteristics of the sample studied and the investigator’s choice of relevant personal attributes. Thus, researchers should avoid the tendency to construe subtypes as more than a theoretical construction. Specific subtyping methods are particularly well suited to certain tasks; different approaches (e.g., two versus four clusters for the same study population) are not necessarily competing representations of “reality.” The usefulness of any particular solution will depend on the investigator’s purpose (for further discussion of this general point, see Del Boca 1994).

A final cautionary note concerns the research sample used in the analyses described in this article. Although the sample is large and diverse with a high proportion of women, and the results are consistent with those from other studies, the profiles that have emerged probably reflect the characteristics used to define the typology as well as the effect of data artifacts such as age. For example, abuse of drugs other than alcohol was more characteristic of younger adults at the time the data were collected, and this pattern may be reflected in the four-group subtype formulation presented here. This result was replicated 15 years later using a large data set from a multisite collaborative study of the genetics of alcoholism (Bucholz et al. in press). Such findings suggest that investigators must be sensitive to contextual factors—such as alcohol use among different age or cultural groups—that may influence the characteristics of particular subtypes obtained in research and that may, in turn, have implications for prevention and treatment efforts. Further research in the typology field will both illuminate such technical pitfalls and uncover the characteristics that most accurately distinguish types of alcoholics from one another.

## Figures and Tables

**Table 1:** Profiles of Type A and Type B Male and Female Alcoholics

Defining Characteristics of Alcoholic Subtypes	Type A	Type B
Risk Factors for Developing Alcoholism		
Familial alcoholism*,**	M < F	M < F
Childhood conduct disorder (e.g., behavioral problems)	M = F	M = F
Measures of personality (McAndrew Scale and MMPT^1^)*	M > F	M = F
Age of onset of problem drinking*	M < F	M = F
Alcohol and Other Substance Use		
Alcohol use (number of ounces per day)*	M > F	M = F
Drinking to relieve negative moods and/or boredom*	M < F	M = F
Severity of alcohol dependence symptoms	M = F	M = F
Tranquilizer use*	M < F	M = F
Polydrug use	M = F	M = F
Chronicity and Consequences of Drinking		
Physical conditions resulting from alcohol use (e.g., liver disease)*	M < F	M = F
Physical consequences of drinking (e.g., hangovers or tremors)*	M < F	M = F
Social consequences of drinking (e.g., job loss or marital problems)*	M > F	M = F
Lifetime alcohol problems (e.g., arrests) (MAST^2^)**	M = F	M > F
Number of years of heavy drinking*,**	M > F	M > F
Psychiatric Symptoms		
Depression symptoms (e.g., sadness)*	M < F	M = F
Antisocial personality (e.g., stealing or fighting)*,**	M > F	M > F
Anxiety symptoms (e.g., nervousness)*	M < F	M = F

* Statistically significant gender differences for type A.

** Statistically significant gender differences for type B.

1 MMPT = Minnesota Multiphasic Personality Test.

2 MAST = Michigan Alcohol Screening Test.

Note: The <, >, and = signs show how men and women compared with each other with respect to each characteristic.

The findings presented are the results of a reanalysis of data presented in Babor et al. 1992.

**Table 2:** Profiles of Four Alcoholic Subtypes

Defining Characteristic	Alcoholic Subtypes			

	Low Risk-Low Severity	Internalizers	Externalizers	High Risk-High Severity
Risk	low	moderate	moderate	high
Alcohol Involvement	low	high	high	moderate
Polydrug Use	low	low to moderate	moderate	high
Alcohol Consequences	low	high	high	moderate
Relief Drinking	moderate	low	high	moderate
Psychiatric Symptoms				
Depression	low	highest	low	high
Antisocial personality	low	low	high	high
Anxiety	low	highest	moderate	high
Gender Composition	mixed (39% female and 28% male)	primarily female (32% female and 11% male)	primarily male (38% male and 7% female)	mixed (22% female and 22% male)
Age	older	older	older	younger

## Footnotes

1 Except where stated, the term “alcoholic” refers in this article to an individual who meets specific criteria for a psychiatric diagnosis of alcohol dependence or abuse. These criteria include the experience of withdrawal symptoms and increased tolerance to the effects of alcohol as well as impaired control over drinking. The term “alcohol abuse” refers to abnormal patterns of drinking that result in detrimental effects on health, social problems, or both.

2 Antisocial personality disorder is a pattern of irresponsible and antisocial behavior beginning early in life and continuing through adulthood.

3 Coping-skills therapy is intended to teach alcoholics specific behavioral skills that help them cope with their urges to drink. Insight-oriented therapy usually involves group sessions and is intended to help alcoholics achieve insights into their reasons for drinking.
